# Factors associated with readmission risk in patients with HIV stratified by BMI: a hospital-based retrospective matched cohort study

**DOI:** 10.1186/s12981-026-00862-9

**Published:** 2026-02-13

**Authors:** Yirong Shi, Qiuxia Deng, Weimei Chen, Liyuan Zhang, Baohong Wu, Suqing Chen, Huiwen Chen, Yun He, Min Wen

**Affiliations:** The Third People’s Hospital of Shenzhen, Shenzhen, 518112 China

**Keywords:** Body mass index, HIV, Readmission, Risk factors, Matched cohort study, Effect modifier

## Abstract

**Background:**

The role of body mass index (BMI) as an effect modifier for readmission risk in people with HIV (PWH) remains underexplored.

**Objective:**

To compare factors associated with two-year all-cause readmission in PWH, stratified by baseline BMI.

**Methods:**

A retrospective matched (1:1 by age/sex) cohort study was conducted at a major public infectious diseases hospital in Shenzhen, China, serving a diverse urban population including migrants and low-income individuals. Adults admitted (Jan–Jun 2020) were stratified into underweight (BMI < 18.5 kg/m², *n* = 80) and normal/overweight (BMI 18.5–<28 kg/m², *n* = 231) groups. Multivariable logistic regression and ROC analysis were performed within each stratum.

**Results:**

The readmission rate was higher in the underweight group (61.3% vs. 46.8%, *p* = 0.023). For underweight PWH, unemployment (adjusted Odds Ratio [aOR] = 8.11, 95% CI 1.82–36.16) and lower nadir CD4 + count (aOR = 0.994 per cell/µL decrease, 95% CI 0.989–0.999) were independent risk factors. For normal/overweight PWH, unemployment (aOR = 2.21, 95% CI 1.12–4.38) and longer time since diagnosis (> 5 vs. 0–1 years, aOR = 4.28, 95% CI 2.02–9.07) were risk factors, while being widowed/divorced was protective (aOR = 0.30, 95% CI 0.10–0.86). Predictive models showed good to excellent accuracy (AUCs: 0.820 and 0.748, respectively).

**Conclusions:**

Risk factors for readmission differ markedly by baseline BMI, confirming its role as an effect modifier. Interventions should be tailored: integrated nutritional and immunological support for underweight PWH, and chronic disease management with attention to social determinants for others.

## Introduction

With HIV becoming a chronic condition, reducing hospital readmissions is crucial for care quality and resource use. Body mass index (BMI) reflects complex health status in PWH, with both underweight and obesity linked to adverse outcomes [1, 2]. However, whether BMI modifies the profile of readmission risk factors is unclear. This study aimed to identify and compare factors associated with two-year readmission in PWH, stratified by baseline BMI. We hypothesized that baseline BMI acts as an effect modifier, meaning that the strength or direction of the association between other risk factors (e.g., socioeconomic, immunological) and readmission risk may differ across BMI categories.

## Methods

### Study design, setting, and participants

This retrospective matched cohort study was conducted at the Infectious Diseases and Immunology Department of Shenzhen Third People’s Hospital in Shenzhen, China. The hospital is a major tertiary care center and the designated hub for HIV/AIDS care and quality control in Shenzhen. Its outpatient clinic manages one of the largest antiretroviral therapy cohorts globally, with a cumulative total of over 20,000 people with HIV in long-term follow-up. The clinic employs a dedicated case management team to support care coordination, counseling, and linkage to services. As the primary referral site in the region, the hospital manages approximately 95–99% of annually reported HIV/AIDS cases in Shenzhen, a megacity characterized by a large mobile migrant population. Therefore, the study cohort is broadly representative of the HIV/AIDS patient population in this high-mobility urban setting.

We included adult patients (age ≥ 18 years) with a confirmed HIV diagnosis who had an index hospitalization (for any cause) between January 1 and June 30, 2020. Inclusion was limited to hospitalizations where HIV infection or an AIDS-defining condition was a primary or contributing diagnosis. Exclusion criteria were: (1) documented AIDS-related cognitive impairment; (2) baseline BMI > 28 kg/m² (Chinese obesity cutoff [3]); (3) death during the index admission; and (4) incomplete baseline data.

### Data collection and analysis

Data were extracted from electronic medical records. Collected variables included demographics (age, sex, marital status, employment), clinical characteristics (time since HIV diagnosis, baseline BMI, nadir CD4 + count, initial viral load), and psychosocial scores. Nutritional risk was assessed using the Nutritional Risk Screening 2002 (NRS-2002) tool, with a score ≥ 3 indicating risk [4]. Anxiety and depression symptoms were assessed using the Hospital Anxiety and Depression Scale (HADS), with a subscale score ≥ 8 indicating clinically significant symptoms [5]. Unemployment was defined as no stable employment for more than six consecutive months prior to the index admission. The primary outcome was two or more all-cause hospitalizations within 24 months after the index discharge.This threshold was chosen to capture recurrent, potentially preventable admissions over a clinically relevant mid-term period, aligning with common readmission quality metrics.Readmission was determined based on objective hospital admission records in the electronic medical system. Standardized institutional criteria for defining a hospital admission (e.g., formal inpatient registration with an admission note) were applied uniformly across all patients. No subjective physician judgment was involved in determining whether a readmission event had occurred, thereby minimizing potential bias.

Patients with readmission were matched 1:1 by age and sex to controls (single admission) using propensity score matching. The matched cohort was stratified into Underweight (BMI < 18.5 kg/m²) and Normal/Overweight (BMI 18.5–<28 kg/m²) groups. The normal-weight and overweight groups were combined for analysis because preliminary analyses showed similar readmission rates and risk factor patterns between these two subcategories, and this grouping enhanced the statistical power for the primary comparison against the underweight group. Statistical analyses were performed separately for each stratum. Multivariable logistic regression models were built with readmission status as the outcome. Variables with *p* < 0.10 in univariate analysis were candidates; age and sex were forced into final models regardless of univariate significance. Backward stepwise elimination derived parsimonious models. Receiver operating characteristic (ROC) curves assessed the predictive performance of the final models. Analyses were performed using SPSS software (version 26.0) and R software (version 4.2.0).

## Results

**Fig. 1:**
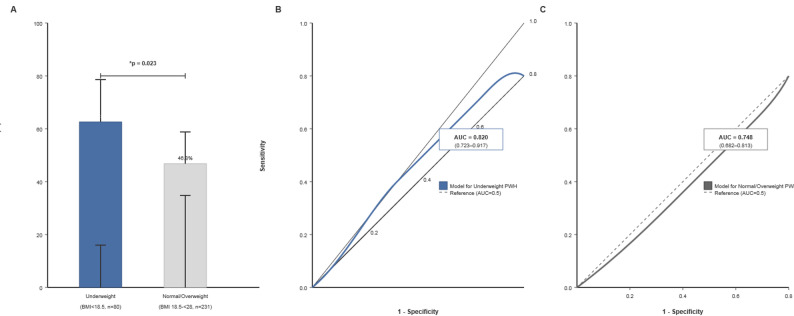
Readmission Rates and Prediction Model Performance Stratified by Baseline BMI.** A** Comparison of two-year all-cause hospital readmission rates between Underweight (BMI < 18.5 kg/m², *n* = 80) and Normal/Overweight (BMI 18.5-<28 kg/m², *n* = 231) people with HIV (PWH). Error bars represent 95% confidence intervals. The difference is significant (χ² test, *p = 0.023).** B** Receiver Operating Characteristic (ROC) curve for the readmission prediction model in Underweight PWH. The model includes unemployment status and nadir CD4 + T-cell count (adjusted for age and sex). The area under the curve (AUC) with 95% confidence interval is shown.** C** ROC curve for the readmission prediction model in Normal/Overweight PWH. The model includes employment status, time since HIV diagnosis (> 5 years), and marital status (widowed/divorced) (adjusted for age and sex). The AUC with 95% confidence interval is shown. In both B and C, the diagonal dashed line represents an AUC of 0.5 (no discriminative power)

The final cohort included 311 patients: 80 underweight and 231 normal/overweight. The overall readmission rate was 49.5% (154/311), significantly higher in the underweight group (61.3% vs. 46.8%, *p* = 0.023) (Fig. 1A).

Key characteristics and bivariate comparisons between BMI strata are shown in Table 1. The factors independently associated with readmission differed by stratum. In the underweight group, unemployment and lower nadir CD4 + count were significant risk factors. In the normal/overweight group, unemployment and longer time since diagnosis (> 5 years) were risk factors, while being widowed/divorced was protective. Other variables (e.g., education, transmission route, NRS-2002 score, HADS scores) were not significant in the final multivariable models and are omitted from the table for brevity.

**Table 1:** Bivariate comparison of characteristics between low BMI (Underweight) and Non-Low BMI (Normal/Overweight) people with HIV, and key multivariable analysis results by stratum

Variable	Underweight (BMI < 18.5) *n* = 80	Normal/overweight (BMI 18.5-<28) *n* = 231	*p*-value (Between Strata)	Multivariable aOR (95% CI) for readmission within stratum
Demographics				
Age (years), Mean ± SD	35.1 ± 11.9	38.6 ± 10.3	0.015	Adjusted for in models
Male Sex, n (%)	69(86.3)	215 (93.1)	0.085	
Marital Status, n (%)			0.045	
Married/Cohabiting	42 (52.5)	154 (66.7)		
Single	32 (40.0)	64 (27.7)		
Widowed/Divorced	6 (7.5)	13 (5.6)		Normal/Wt: 0.30 (0.10–0.86)
Employment Status, n (%)			0.001	
Unemployed, n (%)	24 (30.0)	54 (23.4)		Underweight: 8.11 (1.82–36.16) Normal/Wt: 2.21 (1.12–4.38)
Employed	56 (70.0)	177 (76.6)		
Education Level, n (%)			0.789	
High school or below	45 (56.3)	126 (54.5)		
College or above	35 (43.8)	105 (45.5)		
Clinical characteristics				
Time since Dx, n (%)			0.008	
0–1 year	21 (26.3)	103 (44.6)		Ref.
2–5 years	40 (50.0)	69 (29.9)		
> 5 years	19 (23.7)	59 (25.5)		Normal/Wt: 4.28 (2.02–9.07)
Nadir CD4, cells/µL (Mean ± SD)	171.2 ± 138.1	283.8 ± 279.1	< 0.001	Underweight: 0.994 (0.989–0.999)*
Initial Viral Load (log10), Mean ± SD	4.5 ± 1.0	4.3 ± 1.1	0.211	
NRS-2002 Score ≥ 3, n (%)	51 (63.8)	95 (41.1)	< 0.001	
HADS-A Score ≥ 8, n (%)	22 (27.5)	50 (21.6)	0.342	
HADS-D Score ≥ 8, n (%)	18 (22.5)	40 (17.3)	0.373	
Transmission Route, n (%)			0.654	
MSM	43 (53.8)	136 (58.9)		
Heterosexual	34 (42.5)	87 (37.7)		
Other/Unknown	3 (3.8)	8 (3.5)		
Outcome				
Readmitted, n (%)	49 (61.3)	108 (46.8)	0.023	

*BMI* body mass index,* aOR* adjusted odds ratio,* CI* confidence interval,* Dx* diagnosis; Normal/Wt, Normal/Overweight group; NRS-2002, Nutritional Risk Screening 2002; HADS-A/D, Hospital Anxiety and Depression Scale-Anxiety/Depression subscale; MSM, men who have sex with men. P-values for between-strata comparisons use t-test (continuous) or Chi-square test (categorical). Multivariable logistic regression models were constructed separately for each BMI stratum, adjusted for age and sex. Only variables retained in the final significant models are shown with aOR (95% CI). *Per 1 cell/µL decrease. Other variables assessed (e.g., ART duration) showed no significant independent association in the final models for either stratum

The prediction model for underweight PWH showed excellent discrimination (AUC = 0.820, 95% CI 0.723–0.917) (Fig. 1B). The model for normal/overweight PWH showed good discrimination (AUC = 0.748, 95% CI 0.682–0.813) (Fig. 1C).

## Discussion

### This study confirms that baseline BMI is a key effect modifier for readmission risk in PWH

The profile of independently associated factors differed substantially between BMI strata.Underweight PWH faced higher readmission, driven by socioeconomic vulnerability (unemployment) and advanced immunosuppression (low nadir CD4+). This highlights the need for integrated nutritional and immunological support post-discharge [6]. For normal/overweight PWH, risks were linked to chronicity of HIV infection and unemployment, aligning with challenges of aging with HIV and comorbidities [7]. The protective association observed for being widowed/divorced in the normal/overweight group should be interpreted with caution and may reflect unmeasured social dynamics, health-seeking behaviors, or residual confounding rather than a direct causal effect; this finding warrants further investigation [8].

### BMI as both a modifier and an outcome

While we treated baseline BMI as an effect modifier in this study, we acknowledge the potential for a bidirectional relationship. HIV infection and its treatment can influence weight and body composition, while baseline nutritional status may affect disease progression and resilience. Our study design, which captures BMI at a single point preceding the readmission window, strengthens its conceptual role as a modifier of subsequent risk. Future longitudinal studies with repeated BMI measurements are needed to disentangle these complex temporal relationships.

### Clinical and public health implications

The consistent identification of unemployment as a risk factor across both strata underscores the profound impact of social determinants of health (SDoH) on clinical outcomes. Our findings advocate for the adoption of BMI-stratified risk assessment in clinical practice. Tailored interventions are recommended: for underweight PWH, integrated nutritional and immunological support programs are critical; for normal/overweight PWH, care should focus on proactive comorbidity management combined with systematic screening for SDoH to address root causes of vulnerability [9].

## Strengths and limitations

This study has several strengths, including the use of a large, well-characterized cohort from a major HIV referral center, which enhances the generalizability of findings in similar urban settings, and a matched design that controlled for key demographic confounders. However, several limitations must be considered when interpreting the results. First, the single-center retrospective design may limit generalizability to other regions or healthcare systems and carries an inherent risk of residual confounding. Second, the modest sample size of the underweight group may affect the precision of the risk estimates in that stratum. Third, by excluding PWH with obesity (BMI ≥ 28 kg/m²), our study does not cover the full BMI spectrum; this group merits specific investigation in future research. Fourth, data on the primary reasons for index admission or readmission were not systematically analyzed. While the all-cause readmission outcome is clinically relevant, differences in causes of hospitalization across BMI groups could provide valuable mechanistic insights. Fifth, we did not assess changes in BMI during the follow-up period, which could occur due to disease progression, therapeutic interventions, or lifestyle changes. To address these limitations, future prospective, multi-center studies with longitudinal data collection, including detailed reasons for hospitalization and serial BMI measurements, are needed to validate our findings and further elucidate the causal pathways.

## Conclusion

Risk profiles for hospital readmission differ significantly by baseline BMI in PWH. Personalized, BMI-stratified care pathways are recommended to effectively reduce preventable readmissions.

## Data Availability

The data used to support the findings of this study are available from the corresponding author upon reasonable request.
